# Nutrition Security and Homestead Gardeners: Evidence from the Himalayan Mountain Region

**DOI:** 10.3390/nu17152499

**Published:** 2025-07-30

**Authors:** Nirmal Kumar Patra, Nich Nina, Tapan B. Pathak, Tanmoy Karak, Suresh Chandra Babu

**Affiliations:** 1 Department of Agricultural Extension Education, School of Agricultural Sciences (SAS), Nagaland University, Medziphema 797106, Nagaland, India; 2 Department of Civil and Environmental Engineering, UC Merced, Merced, CA 95343, USA; 3 Department of Soil Science, School of Agricultural Sciences (SAS), Nagaland University, Medziphema 797106, Nagaland, India; 4 Food Policy Research Institute (IFPRI), Washington, DC 20005-3915, USA

**Keywords:** homestead garden (HG), knowledge and perception (K&P), knowledge and perception index (KPI), macronutrient, micronutrient, nutrition security, policy analysis

## Abstract

**Background**: Addressing undernutrition and malnutrition requires a multi-pronged approach targeting different populations with appropriate interventions. Knowledge and perception (K&P) of Individuals and communities about nutrition to human health relationship/continuum is a prerequisite for addressing malnutrition in rural and mountain communities. Assessing K&P is essential for developing strategic interventions to up-scaling K&P of communities and achieving nutrition security. Homestead gardens are a proven intervention for achieving nutrition security for all family members of gardeners. **Methods**: This paper includes homestead gardeners from the Himalayan Mountain Region (HMR) as respondents. We developed a scale to assess the K&P of respondents, based on ratings from 20 judges. A total of 134 issues/items have been retained in the scale from macronutrients, micronutrients, minerals, and vitamins. A framework has also been developed and adopted for the study. A knowledge and perception index (KPI) has been developed based on the respondents’ responses. We have reviewed and analysed the national policy interventions for augmenting the K&P of the study community to achieve nutrition security. **Results**: The nutrition K&P of respondents are inadequate and far from the desirable level. Policy review and analysis indicate that the creation of K&P in the community to contribute to self and family nutrition security was previously highly neglected. **Conclusions**: The policy process of national, state, and county/district-level development sectors in developing countries under the HMR may take the initiative to ensure self-nutrition security by creating K&P of the community on nutrition issues. The designed scale is prudent requires testing and validation for measuring farmers’ K&P on nutrition, which may be adopted in future studies and policymaking not only nationally but also from an international perspective.

## 1. Introduction

Malnutrition, hunger, and food insecurity remain among the most significant global challenges, affecting billions of people worldwide [1,2,3]. Despite decades of progress, the issue persists and is now gradually worsening. According to the FAO, as of 2022, between 691 and 783 million people are undernourished, representing approximately 10% of the global population. Asia, Africa, and Latin America have experienced a rising trend in severe food insecurity in recent years. These alarming figures reflect a troubling reversal of long-term improvements in food security [4]. Malnutrition not only affects physical health but also undermines the immune system, socio-economic equity, and overall development [5,6]. The global food insecurity crisis requires urgent and coordinated efforts to address its root causes. International and regional initiatives must prioritise building resilient food systems, enhancing access to nutritious food, and addressing the socio-economic disparities driving hunger in these vulnerable regions. Without decisive action, the number of people facing chronic hunger is likely to continue rising, further undermining global development goals.

Mountain populations face significant challenges, including stress, hazards, and poverty, contributing to heightened food and nutrition insecurity [7]. In 2024, all eight countries in the Himalayan Mountain Region (HMR), excluding China, were classified as having serious to moderate hunger levels on the Global Hunger Index (GHI). The calculation of GHI includes four components, namely, undernourishment, child stunting, child wasting and child mortality [8]. An adequate supply of food with sufficient content of nutrients (macronutrients and micronutrients) is the only alternative to improve the existing level of the index (GHI). Alarmingly, India’s standing among undernourished nations has shown little improvement over the past five years [9,10]. Malnutrition remains a critical issue, particularly in rural India, where it is more prevalent compared to urban areas [11]. Its intergenerational consequences are severe, as highlighted by Wells et al. [6], who emphasised the erosion of socio-economic equity and overall development caused by malnutrition. Currently, 35.70% of Indian children under five years are underweight, 38.40% are stunted, and 21.00% are wasted [12]. In Arunachal Pradesh, data from 2015–2016 reveal that nearly one-third of children under five years are stunted, 17.00% are wasted, 19.00% are underweight, and 5.00% are overweight [13]. Vulnerable populations, including children and pregnant women in low-income regions, bear the brunt of these impacts, amplifying existing inequalities and intergenerational cycles of malnutrition. Addressing malnutrition in the face of climate change requires a multi-faceted approach, integrating sustainable agricultural practices, climate-resilient food systems, and targeted nutrition programs to build resilience in affected communities. Although there have been improvements since previous surveys, child malnutrition remains a pressing issue both in the region and across India. Multiple factors contribute to malnutrition, including inadequate access to nutritious food. Tackling this issue requires a holistic approach involving targeted interventions and redefined goals within nutrition programs to meet the diverse needs of affected populations [14].

Recognising the vital role of vegetables in ensuring food and nutrition security is increasingly important [15]. Insufficient consumption of fruits and vegetables contributes to malnutrition in developing regions, leading to deficiencies in essential vitamins, minerals, and dietary fiber [16]. For low-income populations in countries such as Bangladesh, India, Pakistan, and Zimbabwe, purchasing the recommended servings of fruits and vegetables can account for half of their daily income [17]. Dark green leafy vegetables are particularly costly, being 5–9 times more expensive as a calorie source than staple cereals in Asia [18]. Homestead gardening, which is a production sub-system designed to produce items primarily for household consumption [19] offers a sustainable solution, enhancing food security and combating malnutrition by providing diverse, nutrient-rich diets at low costs [20,21,22]. This integrated system of cultivating crops and raising livestock supports families with essential food items and resources like organic manure and draught power while improving their nutritional status and quality of life [23,24,25]. As a long-standing component of food systems in developing countries, homestead gardening plays a critical role in regions such as Arunachal Pradesh under the HMR [26,27,28].

One of the primary reasons for poor nutrition is the lack of knowledge and perception (K&P) about nutrition among people. In this study, awareness means knowing about the existence of an innovation, idea, concept, practice and object, and knowledge means the ability to recall with a preliminary understanding about an innovation, idea, concept, practice, and object, whereas perception denotes the ability of perceiving, understanding, and interpreting an innovation, idea, concept, practice, and object. This issue persists globally, regardless of whether food supplies are abundant or scarce. Undernutrition and deficiencies in vitamins and minerals coexist worldwide. Achieving individuals’ nutritional well-being requires an ample supply of high-quality food and a comprehensive understanding of a healthy diet [29]. Assessing nutritional knowledge is crucial in nutrition research, as it enables the development of effective policies and programs that promote healthier eating habits [30]. Consequently, this contributes to enhancing the overall knowledge of nutrition within communities.

According to the existing literature, there is a lack of suitable tools or methodologies for assessing and quantifying the level of K&P among farmers, homestead gardeners, and poultry keepers regarding nutrition, nutrition security, and the correlation between specific livelihood activities and nutrition security. In developing countries, agriculture and allied activities are primarily concentrated in rural areas, playing a vital role in ensuring food and nutrition security at the national level. However, despite this, malnutrition is disproportionately higher in rural areas (with stunting exceeding 50.00% and wasting at 21.00%) compared to urban areas (with stunting at 40.00% and wasting at 17.00%) [31].

Therefore, growers and farmers must have a deep understanding of the nutritional availability within their farm products and the diverse nutritional needs required for maintaining health. Which will support in addressing the complex relationship between ensuring food and nutrition security at a national level and combating malnutrition. It is assumed that addressing rural and national malnutrition can be more effectively achieved by equipping growers with K&P of nutrition. To this end, a unique research framework has been developed and applied to assess homestead gardeners’ nutrition K&P, which can also be adopted to gauge the nutrition K&P of other crop growers and livestock keepers in any place/country. Further, we have extensively analysed the nutrition security policies and malnutrition eradication initiatives implemented by the Government of India since independence in 1947. Our research outcomes and policy analysis will be pivotal in formulating tailored policymaking for all countries (and their respective states) to address malnutrition and achieve comprehensive nutrition security effectively. The present study has the following objectives: (i) to delve into understanding how homestead gardeners perceive and understand nutrition, as well as how they contribute to ensuring nutrition security, (ii) to explore the correlation between social and economic attributes of individuals and their understanding and perspective as homestead gardeners, and (iii) to review/analyse the government of India’s policies for the up-scaling of K&P of individuals on nutrition security to address the nutritional needs of the population. The following null hypothesis was formulated for the testing: H_0_1 = respondents’ socio-economic characteristics have no association with their nutrition knowledge and perception. Accordingly, relationship studies (correlation and regression analysis) were adopted to evaluate and test the hypothesis.

This study offers fresh and impactful insights to the existing body of literature, addressing important gaps, such as, socio-economic profile and nutrition security of rural and mountain people, how to address and achieve the nutrition security of homestead gardeners and farmers through the up scaling of knowledge and perception of growers about nutrition, sources and importance of nutrition for human health. Further, this study is going to enrich the policy mechanism to achieve nutrition security through the individual and societal contributions in the augmentation of knowledge and perception of individuals and communities. Homestead garden may play a significant role in achieving nutritional security in rural and mountain areas.

For instance, the study developed a new scale to assess the K&P of homestead gardeners regarding nutrition, which was tailored for evaluating macronutrients, micronutrients, vitamins, and minerals. This is not extensively covered in existing tools. The study focused on homestead gardeners in the Himalayan Mountain Region. This region has a significantly underrepresented population, which provided insights specific to this geographically challenging and socio-economically diverse area. These factors make the study a novel contribution to both academic research and practical policymaking in the domain. This paper is structured as follows: Section 1 introduces the research, while Section 2 outlines the conceptual framework and methodology employed. Section 3 presents the results, discussion, and an analysis of India’s nutrition policy. The paper concludes with final remarks and recommendations in the last section.

## 2. Methodology

A conceptual framework and knowledge and perception index (KPI) have been developed to complete the study and rigorously assess the respondents’ K&P levels. A clear and direct relationship has been established between the socio-economic variables and the respondents’ K&P levels. The study’s conceptual framework and methodology are poised to be replicated in all the countries under the HMR and other developing and underdeveloped nations. Additionally, in-depth policy analysis on nutrition security within the country can serve as a valuable resource for nutrition policymakers globally, aiding them in formulating effective policy documents that reflect the community’s nutritional perception and the prevailing policy landscape. The following section provides a detailed description of the study area, outlines the conceptual framework used in the study, and discusses the research methods employed to conduct the research.

### 2.1. Profile of the Study Area

The Himalayan Mountain Region (HMR) is extended from 15.95° to 39.31° N latitude and 60.85° to 105.04° E longitude across eight South Asian countries: Afghanistan, Bangladesh, Bhutan, China, India, Myanmar, Nepal, and Pakistan [32,33]. It is extended around 3000 km in length and 250–300 km in width, with a geographical area of around 7,550,000 km^2^ [34]. Around 70% of the people continue agriculture-based livelihoods [35,36] and the maximum number of people are maintaining homestead gardens. Out of the eight countries, India borders all the other countries. Accordingly, India and one of its states (Arunachal Pradesh) under the HMR were selected for the present study.

**Figure 1:**
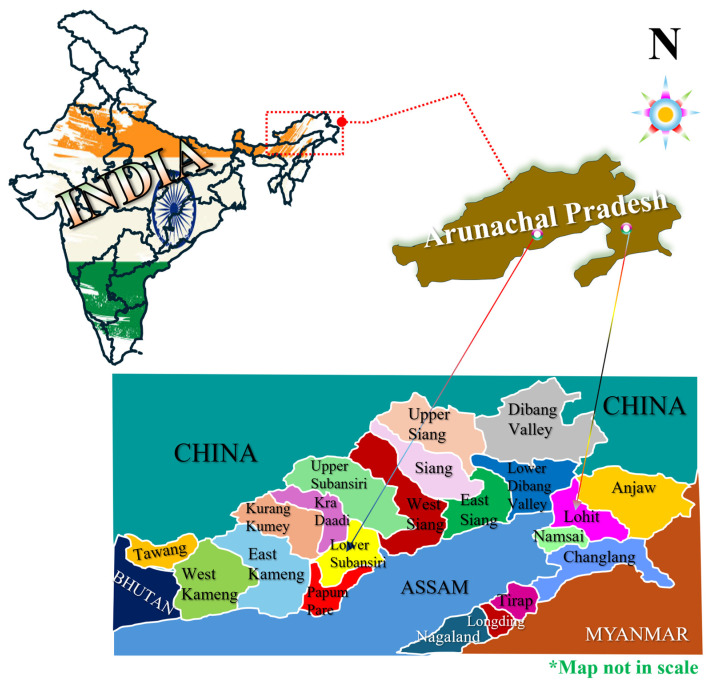
Location maps of the study areas (Lohit and Lowe Subansiri districts) of Arunachal Pradesh, India.

The study took place in Arunachal Pradesh, India, located between 26.28° N and 29.30° N latitude and 91.20° E and 97.30° E longitude. It shares a border with three HMR countries (China, Myanmar, and Bhutan). Hence, the state has been deliberately chosen for the study. A significant portion (29.00%) of the children under the age of five years are stunted in growth [13]. The study focused on the Lower Subansiri district, located at 27.61° N latitude and 93.50° E longitude, and Lohit district, situated at 27.84° N latitude and 96.19° E longitude (Figure 1). These two districts were chosen due to their significant prevalence of malnourished children under five years of age, as indicated by three anthropometric indices of nutritional status: height-for-age, weight-for-height, and weight-for-age [13]. Furthermore, in this study, six villages were purposively selected from two blocks, with one block chosen from each district.

### 2.2. Conceptual Framework

Homestead gardens (HGs) offer a sustainable solution to malnutrition, undernutrition, and micronutrient deficiencies by providing the opportunity to grow diverse and nutrient-rich crops right at home [27,28]. In this study, we adopt Ninez’s definition of an HG [18] as a production sub-system designed to produce items primarily for household consumption. We acknowledged the potential for improving the quality of life for farmers by bolstering local food and nutrition security at a modest cost. One of the leading causes of malnutrition in the population is an insufficient understanding of nutrition and its perception, and addressing this understanding gap will help to reduce malnutrition [29] (please consult the graphical abstract for a schematic view of the conceptual framework).

Based on the literature review, it appears that there are currently no appropriate tools or methods available to measure and quantify the K&P level of the homestead gardeners, fruit growers, crop growers, and livestock keepers regarding nutrition and nutrition security, as well as the interaction between the specific aspects of livelihood components (such as homestead gardening, fruit growing, crop growing, and livestock keeping) and nutrition security. Most existing tools and scales focus on nutrients and their impact on human health. In the HMR’s countries, agriculture and related activities are mainly concentrated in rural areas, where all food items are produced to ensure food and nutrition security. However, malnutrition is more severe in rural areas (stunting: >50.00%; wasting: 21.00%) than in urban areas (stunting: 40.00% and wasting: 17.00%) [13,31]. This poses a paradox: those responsible for ensuring the country’s food and nutrition security suffer from malnutrition and hidden hunger.

Among various reasons, a lack of nutrition K&P is the primary reason for this situation [29]. This is further exacerbated by lower literacy rates in rural (66.77%) compared to urban (84.11%) areas [37]. Regarding the previously mentioned paradox of simultaneously being responsible for ensuring a nation’s food and nutrition security while also contending with malnutrition, we hypothesised that addressing rural and national malnutrition could be straightforward if growers and farmers possessed K&P of the nutritional availability in their farms and the specific nutrient requirements for good health. As a result, we have formulated a research concept and designed a research framework (please consult the graphical abstract) to assess the K&P of homestead gardeners.

The framework (as illustrated in the graphical abstract) outlines the method for assessing K&P levels of homestead gardeners on nutrition and nutrition security, as well as the perceived role of HGs in nutrition security through its components. The study aims to support and influence policy decisions to address malnutrition and achieve nutrition security at both local and national levels.

### 2.3. Respondents

We intentionally selected skilled and experienced homestead gardeners (mean experience: 14.12 years) hailing from specific districts of Arunachal Pradesh, India, to serve as key informants. The respondents demonstrated a high level of proficiency in establishing and caring for HGs. Notably, these individuals were meticulously chosen based on their outstanding maintenance of HGs during the data collection period. To ensure diversity, 120 (60 from each district) homestead gardeners were purposively chosen.

### 2.4. Nutrition K&P Assessment

After a thorough review, 134 specific items/statements were carefully selected and incorporated into the preliminary interview schedule. These items covered three main dimensions: macronutrients, micronutrients (vitamins and minerals), and nutrient contents in food items, aiming to assess the K&P of the respondents. Each item/statement/question was evaluated on a three-point continuum: highly relevant, relevant, and not relevant. The draft interview schedule, including all the items, was then sent to 20 judges for their assessment. These judges included Agricultural University professors, scientists, and faculties, each holding a Ph.D. in agricultural and allied disciplines. Subsequently, the judges’ evaluations were used to finalise the items, with all items judged as highly relevant or relevant being retained. In contrast, those receiving a judgment that was not relevant were excluded from the final schedule.

Following the assessment by the judges, a set of 134 items/statements/questions were retained to evaluate the K&P of individuals regarding nutrition. Additionally, a comprehensive evaluation was conducted, encompassing 35 aspects, including macronutrients, micronutrients, and the availability of nutrients from various commonly consumed food sources. Each aspect was thoroughly addressed, with a minimum of three associated issues. For analysis, the items/statements were assigned a score of 1 or 0 for “yes/aware” or “no/not aware,” respectively. This approach resulted in formulating a knowledge and perception index (KPI) to quantify the respondents’ comprehension of nutrition. With 134 items considered, the potential score ranged from 0 to 134. The resulting index value provided valuable insight into each respondent’s understanding and perception of nutrition’s significance in human health. The methodology utilised to establish the KPI is both intricate and thorough, as follows:

$$Knowledge\;and\;percept\;ionindex\;(KPI)=\frac{Total\;score\;obtained\;by\;the\;respondent}{Total\;achievable\;score}\;\times \;100$$

### 2.5. Policy Review/Analysis

Policy review/analysis involves using various methods of inquiry to produce information-based analysis aimed at addressing policy inadequacies or improving policymaking. It is also an initiative used to contribute remedial measures to protect or rectify flawed policies. Policy analysis as a process of scientific evaluation of the impact of past public policies [38]. Further, policy analysis provides methods and tools for assessing whether a policy is appropriate and suitable [39]. In this study, we employed retrospective policy review/analysis to address malnutrition and achieve nutrition security in India. We considered all the major policy initiatives by the Government of India since the country’s independence in 1947, focusing on analysing K&P enhancement interventions in policy initiatives to encourage participation from every citizen in the eradication of malnutrition and achieve nutrition security.

### 2.6. Data Collection

Both primary and secondary sources were utilised. The primary data were gathered through personal interviews using a structured interview schedule to obtain information from the respondents about their understanding and perception of nutrition and its role in human health. The secondary data were obtained from various sources, such as books, journals, magazines, websites, and other relevant materials.

### 2.7. Statistical Tools and Analysis

This study used a statistical analysis framework to examine the K&P of homestead gardeners regarding nutrition. Initially, Cronbach’s alpha test was adopted to assess the reliability and internal consistency of data collection tools. Accordingly, three separate Cronbach’s alpha tests were adopted for the three tools. The Cronbach’s alpha values were 0.929, 0.972 and 0.896. A high Cronbach’s alpha value (>0.80) indicates greater reliability and internal consistency of the tool. After data collection, all information was cleaned, scored, tabulated, and analysed using a combination of descriptive and inferential statistical tools. Descriptive statistics, including frequency, percentage, mean, and standard deviation, were utilised to summarise respondents’ K&P levels across different dimensions, such as macronutrients, micronutrients, and nutrient content in food items. Correlation analysis was conducted to identify the relationships between socio-economic variables (e.g., education, media exposure, income) and respondents’ K&P scores. Regression analysis further explored the impact of these socio-economic factors on KPI. All statistical analyses were conducted using SPSS version 16, a robust software package widely recognised for its capabilities in managing and analysing complex data sets. For hypothesis testing linear regression analysis was used to evaluate relationships among variables with emphasis on R^2^ and *p*-value of the respective variables of the regression model. Careful attention was paid to ensure data integrity throughout the process, including the appropriate handling of missing values and assumption testing.

## 3. Results and Discussion

This section discusses the research findings, following the study’s objectives.

### 3.1. Macronutrients

Macronutrients are essential for sustaining life and providing essential energy. They are needed in larger quantities and include carbohydrates, fats, proteins, and fibres [40]. Based on the data in Table 1, it is evident that 41.67%, 54.17%, 38.33%, and 93.33% of the respondents were aware of carbohydrates, protein, fibre, and fat. Only 10.83%, 15.00%, 2.50%, and 67.50% of them could correctly recall the sources of carbohydrates, protein, fibre, and fats, respectively. Furthermore, a mere 5.83%, 6.67%, 1.67%, and 11.67% of the respondents acknowledged the importance of carbohydrates, protein, fibres, and fats in providing rapid energy, supporting growth, facilitating development, and maintaining overall bodily functions. The data in Table 1 suggests that the respondents lacked knowledge about macronutrients, their sources, and their importance for human health. In line with this, Kris-Etherton et al. [41] emphasised the necessity of nutrition education, even for medical professionals, which supports our findings about the K&P of the respondents regarding macronutrients.

### 3.2. Micronutrients

In small quantities, micronutrients, such as vitamins and minerals, are essential for the body. However, their impact on health is crucial, and a deficiency in any of them can lead to health issues and life-threatening conditions [42,43]. The insufficient intake of essential vitamins and minerals, known as micronutrient deficiencies, is widespread and often overlooked in discussions on food security [44]. It is universally acknowledged that a comprehensive assessment of a country’s food security must encompass not only macronutrients but also micronutrients and overall nutrition security [5].

According to the data presented in Table 2, 93.33%, 80.00%, and 88.33% of the respondents demonstrated awareness of vitamin A, vitamin B, and vitamin C, respectively. Additionally, 45.00%, 18.33%, and 49.17% of the respondents could accurately identify the sources of vitamins A, B, and C, respectively. Furthermore, 44.17%, 13.33%, and 46.67% of the respondents provided a rationale for the necessity of vitamins A, B, and C in the human body (Table 2).

Table 2 shows that 66.67%, 35.00%, and 19.17% of the respondents knew about vitamin D, K, and E, respectively. On the other hand, 13.33%, 4.17%, and 1.67% of the respondents could recall the sources of vitamin D, E, and K, respectively. At the same time, a mere 12.50%, 4.17%, and 1.67% could articulate the necessity of vitamin D, vitamin E, and vitamin K, respectively, for the human body.

In a study conducted by Alamoudi et al. [45], it was revealed that respondents showed a significant lack of knowledge of vitamin D deficiency. Saini and Hasan [46] found that 64.20% of respondents from urban areas of Bikaner, Rajasthan, India, used multivitamin supplements. Regarding the sources of vitamins, Patil et al. observed that 85.00% of the respondents, who were pharmacy students, were knowledgeable about the sources of vitamin C, and 45.00% of them were unaware of the health benefits associated with vitamin C [47].

The study (Table 3) reveals that 39.17%, 32.50%, and 20.83% of the respondents were familiar with the Ca, Fe, and I, respectively. Further, only 5.00% each and 2.50% of the respondents were able to accurately recall the sources of Ca, Fe, and I, respectively, and a mere 6.67%, 5.00%, and 0.83% could provide a valid explanation for the importance of Ca, Fe, and I, respectively, in the human body. Approximately 18.33%, 16.67%, 16.67%, 15.00%, and 11.67% of the respondents had some prior knowledge about Zn, Na, K, S, and Mn, respectively. Furthermore, 15.83% of the respondents had previously heard about Cl^−^, Cu, Mg, and P, while 84.17% had not been exposed to information regarding these essential elements.

The study’s findings highlight a significant lack of awareness among the general population regarding the crucial role of essential mineral nutrients in maintaining human health. According to [48], even health science students exhibited insufficient knowledge about the importance of minerals in promoting health and preventing diseases. Furthermore, the necessity for patients undergoing maintenance dialysis to better understand dietary phosphorus requirements for optimal health was emphasized [49]. Similarly, Pafili et al. [50] indicated low levels of phosphorus nutritional knowledge among haemodialysis patients and renal nurses in their research.

### 3.3. Exploring Nutrient Content in Commonly Grown/Produced Foods

The diversification of crops in HGs (or farms) directly impacts nutrition security and families’ livelihood [51,52,53]. Therefore, it is essential to understand the K&P of homestead gardeners regarding the nutrient content in commonly grown or used crops to draw valid conclusions.

The nutritional value of crops and agro-ecology directly ensures nutrition security [54]. While cereal-based diets are insufficient in providing micronutrients, legumes, animal-based foods, vegetables, and fruits are good sources of proteins, fats, and micronutrients [55]. Table 4 shows the distribution of respondents based on their K&P of nutrient contents in commonly grown and used cereals (rice and maize). Around 65.00% and 36.67% of the respondents, respectively, were aware that rice and maize are rich sources of carbohydrates, but only a small portion (3.30% and 7.50%) recognised rice and maize as moderate sources of protein, and merely 0.83% and 1.67% identified rice and maize as sources of vitamins. Interestingly, all respondents were unaware that rice has low levels of fats, minerals, and fibres. However, 1.67% recognised maize as a source of minerals.

The *Leguminosae* family plays a crucial role in providing 33.00% of dietary protein, as well as being a source of carbohydrates, fibre, minerals, vitamins, and various phytochemicals [56,57]. The findings show that 63.33% of respondents were aware that legumes/pulses/dals are low in fats, while 41.67% correctly identified them as rich sources of protein. Additionally, all respondents were unaware of the carbohydrates, minerals, fibre, and vitamins content in legumes/pulses/dals. Regarding nuts, only 11.67% of participants correctly identified them as a rich source of fats, and 8.33% recognised them as a source of vitamins. Furthermore, all participants were unaware that nuts were a source of carbohydrates, minerals, protein, and fibre, as indicated in Table 4. Concerning this, Cap et al. (2022) viewed that the nutrition and dietary strength of nuts were less emphasized [58].

The survey results (Table 4) revealed that 92.50% and 97.50% of the respondents were unaware that oranges are a rich source of minerals and vitamins. Additionally, none of the respondents identified oranges as a source of carbohydrates or a low-protein source. Further, 96.67%, 98.33%, 60.00%, and 94.17% of the respondents were unaware that bananas are a rich source of carbohydrates, minerals, vitamins, and fibre, respectively. Surprisingly, all the respondents were unaware that bananas are also a low source of protein.

In Table 4, the distribution of respondents is presented based on their K&P of the nutrient contents in commonly grown/used vegetables in the study area. The majority (88.34%) of the respondents were unaware that green leafy vegetables are a rich source of minerals. Furthermore, all respondents (100%) did not consider green leafy vegetables as a source of protein, and 95.00% were unaware of their rich fibre content. Only 23.33% of the respondents recognised green leafy vegetables as a rich source of vitamins. Additionally, 11.67% of the respondents recognised green leafy vegetables as a rich source of minerals, 5.00% recognised their richness in fibre, and 76.67% acknowledged their vitamins content.

The research conducted by Tedesco et al. [59] and Abukmeil et al. [60] highlighted the immense potential of potatoes in addressing malnutrition. Shockingly, the study (Table 4) revealed that 80.83%, 95.83%, and 96.67% of the respondents were unaware of the fact that potatoes are a rich source of carbohydrates, vitamins and fibre, respectively. Astonishingly, all participants were unaware that potatoes are low in fats and protein but rich in minerals. Only 6.67% of the participants knew about the high fibre content in bamboo shoots, while a mere 15.00% knew that bamboo shoots are a rich source of vitamins (Table 4).

Maintaining plant-based dietary patterns improves human health [61]. However, the adequate consumption of poultry products plays a crucial role in addressing malnutrition among vulnerable groups in India [62]. The data (Table 4) reveal that only 23.33%, 15.83%, 17.50%, and 22.50% of the respondents knew that milk is a rich source of fats, minerals, protein, and vitamins. Interestingly, 100% of the respondents were unaware that milk also serves as a source of carbohydrates.

Regarding the large portions, 85.83%, 96.67%, 73.33%, and 70.00% of the respondents were unaware of eggs’ high-fat, minerals, protein, and vitamins content, respectively. The survey results indicated that the significant portions, i.e., 81.67%, 31.67%, 5.83%, and 3.33% of the respondents, were knowledgeable about chicken being a low source of fats and a rich source of protein, minerals, and vitamins, respectively. Furthermore, 87.5% and 35.83% of the respondents knew that red meat is a rich source of fats and protein. Surprisingly, none recognised red meat as a rich source of minerals and vitamins, as shown in Table 4.

A low level of K&P is a significant constraint to the adoption of improved practices [63,64,65,66,67,68], and it is not an exception in the adoption of nutrition security measures. Table 5 sheds light on the respondents’ overall K&P of nutrition and its importance in human health. The KPI had a maximum possible score of 100, with achieved scores ranging from 2.23 to 56.71. The mean score was 16.18, with a standard deviation of 11.41. These figures indicate that the overall knowledge levels of the respondents were concerning, as the mean KPI value fell significantly short of 25.00% of the maximum achievable score. Patra and Lianzami [69] and Patra et al. [70] also documented similar findings.

### 3.4. Relationship Study

Nutrition knowledge has an association with the habit of nutritious food intake [71]. The findings (Table 6) revealed that family size, area of house premise, and experience in homestead gardening established a non-significant relationship with the KPI of the homestead gardeners. However, positive and statistically significant (at 1%) correlations were observed between nutrition knowledge (KPI) and various socio-economic variables, viz., education, total land holding, facilities, annual expenditure, annual income, mass media exposure, extension contact, and informal sources. A statistically significant correlation (at 5%) was found between income from the HG and the KPI. Further, homestead gardeners’ age also established a negative correlation with their KPI, which indicated that younger people are superior in nutrition knowledge compared to the elderly. Therefore, the study suggests that enhancements in education, extension support, and mass media exposure directly impact nutrition knowledge. It is important to note that [72] emphasise the partial impact of women’s empowerment on maternal and child nutrition, while Ahmed et al. [73] reported insufficient nutrition knowledge among extension workers.

However, it should still be considered in future scaling-up activities, as it may have implications for understanding nutrition and health issues. Based on the relationship study, the null hypothesis is rejected, and socio-economic factors influence the knowledge level of nutrition security of the homestead gardeners. Results from this paper’s correlation study partially agree with the study of Lalthamawii et al. [74].

In the regression analysis (Table 7), the coefficient of determination (R^2^) provides insight into the extent to which the dependent variables explain the variability in the independent variables. In this model, the explanatory variables account for approximately 69.00% (R^2^ = 0.689) of the variation in nutrition knowledge (the dependent variable). The intercept represents the estimated value of the dependent variable (nutrition knowledge) when all independent variables are zero, with a predicted nutrition knowledge level of 3.620 units. Moreover, education and mass media exhibit statistically significant effects on the dependent variable in this model. Specifically, for every unit increase in the respondent’s education, we anticipate a 1.43-unit increase in nutrition knowledge, and this finding has an alignment with the finding of [75]. In comparison, a 1-unit increase in media exposure is linked to a 2.13-unit rise in nutrition knowledge. Additionally, age demonstrates a near-significant negative effect on nutrition knowledge at 10.00% (*p*-value 0.11), suggesting that as age increases by 1 unit, nutrition knowledge decreases by 0.13 units.

### 3.5. Policy Scenario

In this subsection, we retrospectively review/analyse the status of policy intervention to address malnutrition and achieve nutrition security in India. We adopted Patton and Sawicki’s (1993) Sawicki’s [76] model of policy analysis. Defining and detailing the problem is the first step in policy review/analysis. Based on our research, nutrition security and addressing malnutrition emerged as a recognised problem. To address the problem, people’s participation and contribution are needed. The contribution and involvement of individuals are based on the K&P of the problems and their solutions. Accordingly, K&P of home gardeners about nutrition, malnutrition, nutrition security, and sources of nutrition from commonly consumed food items, fruits, and vegetables and the importance of nutrition to human health is considered a recognised problem.

Establishing evaluative criteria to analyse the recognised problem is the second step. Major policy initiatives by the Government of India and different state governments related to nutrition security in the last 75 years were considered. Nutrition security is well emphasised and accepted by policymakers as a policy agenda in India. For example, Article 47 of the Indian Constitution (The State shall regard the raising of the level of nutrition and the standard of living of its people and the improvement of public health) is concerned about the nutrition security of the citizens of the country [77]. Some of the early nutrition initiatives include the Goitre Control Programme in 1962 [78], the Special Nutrition Programme in 1970–1971; the Balwandi Nutrition Programme in 1970–1971; the Nutritional Anaemia Prophylaxis Programme with Iron and Folic Acid to mother and children in 1970; the Prophylaxis programme against blindness due to Vitamin A deficiency in 1970 [79,80]. All these programmes aimed to increase the supply of nutrients to the target community. However, improved access to nutrition knowledge, awareness creation, and the augmentation of the community’s ability to address malnutrition were relatively less emphasised by the policymaking process.

The introduction of the Integrated Child Development Services (ICDS) in 1975 was a critical juncture in the nutrition security policy scenario. The ICDS emphasised mothers’ and children’s nutrition security at the expense of awareness creation among the citizens, which was less prioritised [34]. The reconstitution of the Food and Nutrition Board in 1990 was also a policy intervention used to achieve nutrition security along with improved food security. Another initiative was the introduction of the National Nutrition Policy in 1993 [81]. It recognised that achieving nutrition security was a multi-sectoral issue and needed the convergence of all stakeholders. Further, it included the awareness creation of citizens through the inclusion of nutrition issues in the school syllabus; however, awareness among general citizens continued to be neglected.

The provision of mid-day meals in school was well recognised in 1956 by the erstwhile Madras presidency (now Tamil Nadu state, India). Later on, in 1995, this school lunch concept was adopted and introduced throughout the nation for school children up to class VIII to ensure education for all, and children’s nutrition security was complimentarily addressed [82]. The National Public Distribution System was introduced throughout the nation in 1997, which also played the role of nutrition security as complimentary to food security [83]. In 2005, the National Rural Health Mission was introduced in the nation to prevent disease [84]. Later on, in the year of 2013, the National Food Security Act was introduced with an emphasis on ensuring food security, which also played a role in reducing the protein/nutrition deficiency of poor citizens. However, the self-contribution of individual citizens to achieve nutrition security through the awareness and perception creation of individuals was neglected [85].

However, after the national food security achievement, India’s government emphasised achieving nutrition security. Accordingly, the National Nutrition Mission was introduced in 2001. Later on, an improved and revitalised programme was introduced throughout the nation as the National Nutrition Mission (also called PM POSHAN Abhiyan) in the year of 2018. The POSHAN Abhiyan programme is still continuing throughout India to secure nutrition security with various objectives. However, the augmentation of individuals’ perception of nutrition security through different programmes for self-sufficiency in nutrition security was even neglected in POSHAN Abhiyan [86,87].

It is clear from the present policy review/analysis that the nutrition security of children and mothers was emphasised, and the malnutrition of adults was neglected. But, malnutrition is also a prominent problem in adults [88]. Providing supplementary requirements of nutrients to children and pregnant and lactating mothers may bring some extent of nutrition security. The availability of and access to nutritious food, inappropriate child feeding, and dietary choices of adults are also the reasons for malnutrition [89]. Awareness about the nutritive value of food items has an association with the healthy living of people [90]. Therefore, awareness/perception creation on nutrient contents in the foods and their role in good body and health maintenance is essential for avoiding malnutrition and disease prevention in humans [91]. But, the awareness and perception creation of individuals, parents, and adults have relatively been less ephasised by the policymaking process.

Finally, the next step of policy review/analysis is identifying an alternative policy or proposing a modified policy. We are proposing that adequate knowledge and a strong perception of citizens on malnutrition, nutrition science, and security are needed to combat malnutrition and to achieve nutrition security in India and other countries under the HMR and developing countries.

### 3.6. Insightful Explanation

This study sheds light on critical aspects of nutrition security among homestead gardeners in the Himalayan Mountain Region, revealing significant gaps in K&P and offering pathways for intervention. The findings highlight four key points that contribute to the discourse on food security and nutrition policy.

#### 3.6.1. Knowledge Gaps and Socio-Economic Correlations

The study highlights alarming deficiencies in the K&P of nutrition among homestead gardeners, despite their critical role in food production. While many respondents were familiar with basic nutritional concepts, their understanding of nutrient sources, functions, and health benefits was insufficient. This knowledge gap not only undermines their ability to make informed dietary choices but also limits the potential of their agricultural practices to address malnutrition within their households and communities.

The analysis revealed that socio-economic factors, including education, income, landholding size, and access to media, significantly influence nutrition knowledge. For instance, individuals with higher levels of education and greater media exposure demonstrated better awareness of macronutrients and micronutrients. These findings underscore the importance of addressing structural inequalities, such as limited access to education and information, to improve nutrition outcomes. Targeted interventions, such as community-based education programs and information dissemination through accessible media, can bridge these gaps and empower farmers to leverage their resources effectively.

#### 3.6.2. Homestead Gardening as a Sustainable Solution

Homestead gardening emerged as a practical and sustainable solution to combat malnutrition and food insecurity in rural areas. These gardens provide households with direct access to diverse and nutrient-rich foods, including fruits, vegetables, and livestock products, at a relatively low cost. Beyond ensuring caloric sufficiency, homestead gardens are a critical source of essential micronutrients, which are often lacking in staple-based diets.

This study corroborates existing evidence that homestead gardens contribute to dietary diversity, reduce dependency on external food systems, and promote self-reliance. Additionally, the integrated approach of combining crop cultivation with livestock rearing offers multiple benefits, such as organic manure production and improved livelihoods. Encouraging widespread adoption of homestead gardening through capacity-building programs, subsidies for seeds and inputs, and extension services tailored to local needs could significantly enhance nutrition security in the HMR and other similar regions. In addition, homestead gardening can be cultivated with sustainable practices such as the use of composts, organic materials, etc., to contribute towards more resilient and sustainable food production.

#### 3.6.3. Policy Gaps and Opportunities

A retrospective review of India’s nutrition policies revealed critical gaps in addressing adult malnutrition and fostering community-level awareness. While programs such as the Integrated Child Development Services (ICDS) and National Nutrition Mission have made strides in improving maternal and child nutrition, they fail to engage adults, particularly farmers and gardeners, who are key stakeholders in food production and security.

This study identifies a missed opportunity in leveraging community-level contributions to nutrition security. Policies to date have largely focused on direct interventions, such as food supplementation and healthcare services, without adequately addressing the importance of K&P creation among citizens. Integrating nutrition education into policy frameworks can empower communities to take proactive roles in combating malnutrition. For example, incorporating nutrition awareness campaigns into agricultural extension programs, school curriculums, and public health initiatives could foster a culture of informed decision-making and self-sufficiency.

#### 3.6.4. The Role of Customised Tools and Frameworks

One of the most innovative aspects of this study is the development and application of the Knowledge and Perception Index (KPI). This tailored tool enables a nuanced assessment of nutrition knowledge, offering insights into specific areas where awareness is lacking. By quantifying K&P levels, the KPI provides a replicable model for evaluating the effectiveness of nutrition interventions across different populations and contexts.

Additionally, the study’s conceptual framework offers a structured approach to understanding the interplay between socio-economic factors, agricultural practices, and nutrition outcomes. Policymakers and development practitioners can adopt this framework to design targeted interventions that address the unique needs of specific communities. For instance, it could guide the allocation of resources to high-priority areas, such as providing training on nutrient-dense crop cultivation or improving access to information through digital platforms.

#### 3.6.5. Connection to Climate Change and Resilience

The findings of this study must be contextualised within the broader challenge of climate change, which poses a significant threat to food and nutrition security in vulnerable regions like the HMR. Rising temperatures, rainfall uncertainties, and an increased frequency and intensity of extreme weather events impact agricultural productivity, particularly for small-scale and subsistence farmers. Homestead gardens, with their diversity of crops and adaptive potential, present a sustainable and resilient solution to mitigate these climate-related risks.

By cultivating diverse, nutrient-rich crops that are better suited to changing climatic conditions, homestead gardening can buffer households against food insecurity during adverse environmental events. Furthermore, integrating climate-smart agricultural practices into homestead gardening, such as water conservation techniques, agroforestry, and crop diversification, can enhance resilience and long-term sustainability. Policymakers should recognise the dual role of homestead gardens in improving nutrition and addressing climate vulnerabilities, incorporating them into broader climate adaptation strategies.

#### 3.6.6. The Role of Cooperative Extension Programs

Cooperative extension programs can provide a unique solution-driven opportunity by translating important nutrition and homestead gardening research-based approaches to rural populations in a way that empowers actions. By leveraging local networks and expertise, cooperative extension services can provide targeted education and training to homestead gardeners, equipping them with the skills and knowledge needed to enhance their nutritional practices as well as sustainable gardening practices. These programs can focus on teaching sustainable agricultural methods, promoting nutrient-dense crops, and raising awareness about the importance of balanced diets. Additionally, extension workers can serve as crucial links between policymakers and communities, ensuring that government initiatives are effectively implemented at the grassroots level. Cooperative extension programs can also foster peer learning by organising farmer-to-farmer knowledge exchanges, thereby creating a supportive ecosystem for addressing malnutrition collectively.

## 4. Conclusions

It is evident that huge inadequacies in the nutrition awareness and importance of nutrition for the proper mental and physical health of human beings are persisting in the rural and mountain communities. Further, policy-level deficiency with reference to nutrition awareness creation to achieve self-nutrition security by individual citizens is also recognized. The lack of K&P about nutrition and its significance for human health has several implications for addressing malnutrition. K&P about nutrition is crucial for implementing nutrition-sensitive policies in the country. The limited available literature emphasises the importance of raising public K&P about nutrition security in developing countries under the HMR. It is obvious that the public sector of developing countries is unable to bring nutrition security for the vast population due to various institutional and organisational constraints; it is difficult for the government to reach or directly serve all the citizens. Therefore, the policy process of the developing countries under HMRs may take the initiative to ensure that the self, family, and community level contribution in nutrition security drive through the creation of K&P of the community on nutrition issues.

Therefore, all the concerned departments may take the initiative for the analysis of existing policies and to explore the opportunity to incorporate different activities on the K&P creation of citizens on nutrition security, or may propose new policies. All the departments involved in welfare, health and family welfare, and food and nutrition security programmes may augment the capacity of individuals to achieve self and family nutrition security instead of offering items or subsidised inputs. It is crucial for the Department of Health and Family Welfare to take the initiative to bring the individual’s and family’s nutrition security by involving all citizens. Capacity development of their extension workers is urgently needed to up-scale the K&P of the community and mothers of young children. Basic nutrition education should be incorporated into the primary, higher secondary, and college syllabi. The undergraduate (UG) and postgraduate (PG) syllabus of agriculture, horticulture, and livestock education may be restructured, with an emphasis on mechanisms to accelerate and achieve nutrition security. The Department of Agriculture, The Department of Horticulture, and other allied departments at national and state levels are responsible for promoting the cultivation and consumption of nutrition-rich crops. Accordingly, they may analyse their functionaries’ competency for nutrition extension, and the required capacity strengthening of functionaries needs to be initiated. Also, there is a need to analyse existing policies and modify them, emphasising up-scaling the K&P of farmers, female farmers, and villagers on nutrition security, nutrition-rich crops, and the importance of nutrition to human health. Further, all the concerned departments may emphasise a group approach through women self-help groups (SHGs), male SHGs, Farmer Producer Organisations (FPOs), and growers’ associations, which accelerate the achievement of the target of nutrition security.

However, based on the research findings, it can be concluded that a multi-faced initiative is needed to involve all stakeholders, along with a special emphasis on up-scaling the awareness and perception of individual citizens, which will support addressing the community’s malnutrition. Despite several positive outcomes from the study, it has some limitations, including that it is based on perceived data, which relies on the recall and perception abilities of respondents, and the possibility of bias and error exists. Furthermore, the findings are based on a small sample, which may limit the generalisability of the study outcomes. Accordingly, to overcome the limitations and to augment the robustness of the study outcomes, we are continuing a similar type of research in different areas with a bigger sample size. Similar types of study initiatives from other researchers may accelerate the achievement of nobility in the genre of research. For instance, a study on marginal and small farmers’ ability to address nutrition security through their farming components. It may also be related to the level of nutrition security of different categories of farmers through their farming. Also, assessing the influence of socio-economic factors on the adoption of nutrition-rich farming components in their farming. Or, the influence of socio-economic factors and the degree of achieving nutritional security.

## Figures and Tables

**Table 1:** Distribution of respondents based on their knowledge of macronutrients (N = 120).

Sl. No.	Macro Nutrients	Issues/Items/Questions	Yes		No	
			F ^$^	P ^$$^	F	P
1.	Carbohydrates	Have you heard of it?	50	41.67	70	58.33
		Can you name any source?	13	10.83	107	89.17
		Do you know why it is needed for our body?	7	5.83	113	94.17
2.	Fat	Have you heard of it?	112	93.33	8	6.67
		Can you name any source?	81	67.50	39	32.50
		Do you know why it is needed for our body?	14	11.67	106	88.33
3.	Fibre	Have you heard of it?	46	38.33	74	61.67
		Can you name any source?	3	2.50	117	97.50
		Do you know why it is needed for our body?	2	1.67	118	98.33
4.	Protein	Have you heard of it?	65	54.17	55	45.83
		Can you name any source?	18	15.00	102	85.00
		Do you know why it is needed for our body?	8	6.67	112	93.33

^$^ F = frequency, ^$$^ P = percentage.

**Table 2:** Distribution of respondents based on their knowledge of vitamins (N = 120).

Sl. No.	Vitamins	Issues/Items/Questions	Yes		No	
			F ^$^	P ^$$^	F	P
1.	A	Have you heard of it?	112	93.33	8	6.67
		Can you name any source?	54	45.00	66	55.55
		Do you know why it is needed for our body?	53	44.17	67	55.83
2.	B	Have you heard of it?	96	80.00	24	20.00
		Can you name any source?	22	18.33	98	81.67
		Do you know why it is needed for our body?	16	13.33	104	86.67
3.	C	Have you heard of it?	106	88.33	14	11.67
		Can you name any source?	59	49.17	61	50.83
		Do you know why it is needed for our body?	56	46.67	64	53.33
4.	D	Have you heard of it?	80	66.67	40	33.33
		Can you name any source?	16	13.33	104	86.67
		Do you know why it is needed for our body?	15	12.50	105	87.50
5.	E	Have you heard of it?	42	35.00	78	65.00
		Can you name any source?	5	4.17	115	95.83
		Do you know why it is needed for our body?	5	4.17	115	95.83
6.	K	Have you heard of it?	23	19.17	97	80.83
		Can you name any source?	2	1.67	118	98.33
		Do you know why it is needed for our body?	2	1.67	118	98.33

^$^ F = frequency, ^$$^ P = percentage.

**Table 3:** Distribution of respondents based on their knowledge of minerals (N = 120).

Sl. No.	Minerals	Issues/Items/Questions	Yes		No	
			F ^$^	P ^$$^	F	P
1.	Calcium (Ca)	Have you heard of it?	47	39.17	73	60.83
		Can you name any source?	6	5.00	114	95.00
		Do you know why it is needed for our body?	8	6.67	112	93.33
2.	Chloride (Cl)	Have you heard of it?	19	15.83	101	84.17
		Can you name any source?	0	0	120	100
		Do you know why it is needed for our body?	0	0	120	100
3.	Copper (Cu)	Have you heard of it?	19	15.83	101	84.17
		Can you name any source?	0	0	120	100
		Do you know why it is needed for our body?	0	0	120	100
4.	Iodine (I)	Have you heard of it?	25	20.83	95	79.17
		Can you name any source?	3	2.50	117	97.50
		Do you know why it is needed for our body?	1	0.83	119	99.17
5.	Iron (Fe)	Have you heard of it?	39	32.50	81	67.50
		Can you name any source?	4	3.33	116	96.67
		Do you know why it is needed for our body?	5	4.17	115	95.83
6.	Magnesium (Mg)	Have you heard of it?	19	15.83	101	84.17
		Can you name any source?	0	0	120	100
		Do you know why it is needed for our body?	0	0	120	100
7.	Phosphorus (P)	Have you heard of it?	19	15.83	101	84.17
		Can you name any source?	0	0	120	100
		Do you know why it is needed for our body?	0	0	120	100
8.	Potassium (K)	Have you heard of it?	20	16.67	100	83.33
		Can you name any source?	0	0	120	100
		Do you know why it is needed for our body?	0	0	120	100
9.	Sodium (Na)	Have you heard of it?	20	16.67	100	83.33
		Can you name any source?	0	0	120	100
		Do you know why it is needed for our body?	0	0	120	100
10.	Sulfur (S)	Have you heard of it?	18	15.00	102	85.00
		Can you name any source?	0	0	120	100
		Do you know why it is needed for our body?	0	0	120	100
12.	Zinc (Zn)	Have you heard of it?	22	18.33	98	81.67
		Can you name any source?	0	0	120	100
		Do you know why it is needed for our body?	0	0	120	100

^$^ F = frequency, ^$$^ P = percentage.

**Table 4:** Distribution of respondents based on their knowledge about nutrient content in commonly available items, viz., cereals, legumes, nuts, fruits, and green vegetables, and non-vegetable items, viz., milk, egg, and meat (N = 120).

Sl. No.	Issues/Items/Questions	Aware		Not Aware	
		F ^$^	P ^$$^	F	P
1.	Rice				
	It is a rich source of carbohydrate	78	65.00	42	35.00
	It contains a low amount of fats	0	0	120	100
	It is a good source of minerals	0	0	120	100
	It contains a moderate amount of protein	4	3.33	116	96.67
	It contains a low amount of fibre	0	0	120	100
	It is a good source of vitamins	1	0.833	119	99.17
2.	Maize				
	It is a rich source of carbohydrate	44	36.67	76	63.33
	It contains a low amount of fats	10	8.33	110	91.67
	It is rich in minerals content	2	1.67	118	98.33
	It is a moderate source of protein	9	7.50	111	92.50
	It contains a high amount of fibre	5	4.17	115	95.83
	It contains a high amount of vitamins	2	1.67	118	98.33
3.	Pulse/Legume/Dal				
	It is a rich source of carbohydrate	0	0	120	100
	It contains a low amount of fats	76	63.33	44	36.67
	It is generally high in minerals content	0	0	120	100
	It is a rich source of protein	50	41.67	70	58.33
	It is high in fibre content	0	0	120	100
	It is a rich source of vitamins	0	0	120	100
4.	Nuts				
	Are generally low in carbohydrates	0	0	120	100
	They are a rich source of fats	14	11.67	106	88.33
	They contain a high amount of minerals	0	0	120	100
	They are a rich source of protein	0	0	120	100
	They are a good source of fibre	0	0	120	100
	They are a good source of vitamins	10	8.33	110	91.67
5.	Orange				
	It contains a low amount of carbohydrate	0	0	120	100
	It is a rich source of minerals	9	7.50	111	92.50
	It contains a low amount of protein	0	0	120	100
	It is rich in fibre content	3	2.50	117	97.50
	It is a rich source of vitamins	73	60.83	47	39.17
6.	Banana				
	It is a rich source of carbohydrate	4	3.33	116	96.67
	It contains a low amount of fat	2	1.67	118	98.33
	It contains a high amount of minerals	9	7.50	111	92.50
	It contains a low amount of protein	0	0	120	100
	It is a rich source of fibre	7	5.83	113	94.17
	It is a rich source of vitamins	48	40.00	72	60.00
7.	Green leafy vegetables				
	They are a rich source of minerals	14	11.67	106	88.34
	They are also a source of protein	0	0	120	100
	They are a rich source of fibre	6	5.00	114	95.00
	They contain a high amount of vitamins	92	76.67	28	23.33
8.	Potato				
	It is a rich source of carbohydrate	23	19.17	97	80.83
	It contains a low amount of fat	0	0	120	100
	It contains a high amount of minerals	0	0	120	100
	It contains a low amount of protein	0	0	120	100
	It is high in fibre content	4	3.33	116	96.67
	It is a rich source of vitamins	5	4.17	115	95.83
9.	Bamboo shoot				
	It is a rich source of carbohydrate	0	0	120	100
	It contains a low amount of fat	0	0	120	100
	It is a rich source of minerals	0	0	120	100
	It is a rich source of protein	0	0	120	100
	It contains a high amount of fibre	8	6.67	112	93.33
	It is a rich source of vitamins	18	15.00	102	85.00
10.	Milk				
	It is a source of carbohydrate	0	0	120	100
	It contains a high amount of fats	28	23.33	92	76.67
	It is a rich source of minerals	19	15.83	101	84.17
	It is a rich source of protein	21	17.50	99	82.50
	It is a rich source of vitamins	27	22.50	93	77.50
11.	Egg				
	It contains a high amount of fats	17	14.17	103	85.83
	It is a rich source of minerals	4	3.33	116	96.67
	It is a rich source of protein	32	26.67	88	73.33
	It is a rich source of vitamins	36	30.00	84	70.00
12.	Chicken				
	It has a low-fat content	98	81.67	22	18.33
	It is a rich source of minerals	7	5.83	113	94.17
	It is a rich source of protein	38	31.67	82	68.33
	It is a good source of vitamins	4	3.33	116	96.67
13.	Red meat				
	It contains a high amount of fats	105	87.50	15	12.50
	It has a high minerals content	0	0	120	100
	It is a rich source of protein	43	35.83	77	64.17
	It is a good source of vitamins	0	0	120	100

^$^ F = frequency, ^$$^ P = percentage.

**Table 5:** Distribution of respondents based on overall awareness and knowledge level about nutrition and its importance in human health (N = 120).

Sl. No.	Knowledge Level	F ^$^	P ^$$^	Knowledge Index (KI)			
				Range		Mean	SD
				Possible	Achieved		
1	Very low	29	24.17	0–100	2.23–56.71	16.18159	11.41407
2	Low	43	35.83				
3	Medium	28	23.33				
4	High	20	16.67				
Total		120	100				

^$^ F = frequency, ^$$^ P = percentage.

**Table 6:** Relationship between socio-economic factors and the nutrition knowledge index.

Sl. No.	Socio-Economic Variables	Value ‘r’
1	Age	−0.3496 **
2	Family size	0.0676 NS
3	Education	0.7720 **
4	Total land holding	0.3353 **
5	Area of house premise	0.1667 NS
6	Experience in homestead gardening	−0.0954 NS
7	Facilities	0.3028 **
8	Annual expenditure	0.3838 **
9	Income from homestead gardening	0.1969 *
10	Annual income	0.3431 **
11	Mass media	0.5931 **
12	Extension contact	0.2990 **
13	Informal sources of information	0.2442 **

* Significant at 5%; ** significant at 1%; NS—not significant.

**Table 7:** Regression analysis: between socio-economic variables and nutrition knowledge (KPI) level of homestead gardeners; R^2^ = 0.689; intercept = 3.620; ** = significant at 1%; * = significant at 10%; NS = non-significant.

Variables	Coefficient	SE	T Value of β
Age (yrs.)	−0.135	0.081	−1.660 *
Family size (No.)	−0.012	0.314	−0.039 NS
Education (yrs.)	1.419	0.174	8.147 **
Landholding (in acre)	0.044	0.160	0.274 NS
Area of house premise (in acre)	1.323	1.214	1.089 NS
Experience in homestead gardening (yrs.)	0.199	0.122	1.627 *
Facilities (No.)	0.069	0.870	0.080 NS
Expenditure (Rs.)	0.002	4.6	0.509 NS
Mass media exposure ((No.)	2.11	0.819	2.576 **
Extension contact (No.)	0.598	0.456	1.304 NS
Income from homestead garden (Rs.)	2.1	2.3	0.900 NS

## Data Availability

The original contributions presented in the study are included in the article; further inquiries can be directed to the corresponding author.
